# The Acute Burn ResUscitation Multicenter Prospective Trial 2 (ABRUPT2)

**DOI:** 10.1093/jbcr/irag033.579

**Published:** 2026-04-06

**Authors:** David G Greenhalgh, Robert Cartotto, Sandra Taylor, Jeffrey Fine, David Wallace, Mahmoud Hassouba, Sai Ram Velamuri, Michael A Marano, Julia C Slater, Niknam Eshraghi, Peter Kwan, Lucy Wibbenmeyer, Angela Gibson, Francis V Winski, Jr., James H Holmes, IV, Justin E Dvorak, Jeffrey Claridge, Sarah Fischer, Andrea Munden, John Kubasiak, Dhaval Bhavsar, Tam N Pham, Shevonne S Satahoo, Nicholas A Meyer

**Affiliations:** Shriners Children’s Northern California, Davis, CA; University of Toronto, Toronto, ON; University of California Davis, Sacramento, CA; University of California Davis, Sacramento, CA; Ross Tilley Burn Centre, Toronto, ON; Firefighters Regional One Burn Center, Memphis, TN; USF Health Morsani College of Medicine, Tampa, FL; Cooperman Barnabas Medical Center, Chatham, NJ; The University of Kansas Health System Burnett Burn Center, Kansas City, KS; Legacy Oregon Burn Center, Portland, OR; University of Alberta, Edmonton, AB; University of Iowa Healthcare, Iowa City, IA; University of Wisconsin Burn and Wound Center, Madison, WI; Westchester Medical Center, Monroe, NY; Atrium Health Wake Forest Baptist Burn Center, Winston-Salem, NC; MetroHealth Medical Center, Cleveland, OH; MetroHealth Medical Center, Cleveland, OH; Ascension Via Christi Regional Burn Center, Wichita, KS; University of Florida Health Shands Burn Center, Gainesville, FL; Loyola University Chicago, Maywood, IL; The University of Kansas Health System, Kansas City, KS; University of Washington Harborview Medical Center, Seattle, WA; Miami Burn Center, Miami, FL; Ascension Columbia St Mary’s, Mequon, WI

## Abstract

**Background/Objective:**

Human Albumin Solution (Alb) is widely used in burn shock resuscitation (BSR), even though prospective randomized studies are 20-50 years old. The observational ABRUPT1 study found that Alb lowered resuscitation volumes and improved urine output (UO). ABRUPT2 provides the only modern randomized prospective study comparing Alb to crystalloid. The purpose was to compare BSR using albumin to crystalloids alone. The hypothesis is that Alb reduces fluid volumes compared to lactated Ringer’s (LR) alone for BSR.

**Methods:**

An international multicenter randomized prospective trial comparing 5% Alb to LR alone during BSR, among adults with burns >25% TBSA with >20% TBSA full thickness injury. Patients randomized to Alb received fluids consisting of one-third 5% Alb and two thirds LR, starting between 8-12 hours post burn. Resuscitation was titrated to achieve UO of 0.5-1 mL/kg/hr. For safety or excessive resuscitation volumes, investigators were allowed to crossover patients between assigned study arms in the 1st 48 hr. All analyses are based on intention-to-treat. The primary outcome was the volume of fluid administered in the 1st 24 hours.

**Results:**

The study was halted at 99 subjects (Alb n = 48, LR n = 51) of a planned 400, due to slow enrollment and a 51% crossover rate from LR to Alb in the 1st 48 hr with no Alb to LR crossovers. The overall study population’s mean +/- SD age was 45.9□15.9 years, 80% were male, with 45.7□13.9% TBSA burn, 37.5□14.9% full-thickness burn, 13.3% with smoke inhalation injury, and 97.0% with flame burns. Total fluid administration in mL/kg/% TBSA burn for LR and Alb respectively were 5.21□2.53 versus 3.88□2.13 at 24 hours. Regression models which controlled for age, burn size and inhalation injury found that the LR group had received 150% more fluid in mL/kg/%TBSA burn (*p*=.001) and 210% more fluid (*p*<.001) than the albumin group at 24 and 48 hours, respectively (Figure). I:O ratios at 24 and 48 hours were lower in the Alb group (*p*<.001). LR patients who crossed over to Alb displayed elevated and chaotic I:O ratios characteristic of a “runaway” resuscitation, which stabilized upon initiation of Alb. Alb patients had significantly lower peak lactate and peak intra-abdominal pressure. There were no significant differences between Alb and LR in time to wound healing, Acute Kidney Injury, or mortality.

**Conclusions:**

Administration of Alb at 8-12 hours post burn resulted in a statistically significant and clinically important reduction in fluid resuscitation volumes, improved UO, and lower peak lactate and IAP compared to using LR alone. Alb was also effective in rescuing uncontrolled runaway resuscitations.

**Applicability of Research to Practice:**

Alb is an effective strategy to lower BSR volumes and to rescue patients receiving excessive crystalloid volumes during BSR.

**Funding for the study:**

Department of Defense.

